# Neuropilin-1 promotes the oncogenic *Tenascin-C/*integrin β3 pathway and modulates chemoresistance in breast cancer cells

**DOI:** 10.1186/s12885-018-4446-y

**Published:** 2018-05-05

**Authors:** Adviti Naik, Aida Al-Yahyaee, Nada Abdullah, Juda-El Sam, Noura Al-Zeheimi, Mahmoud W. Yaish, Sirin A. Adham

**Affiliations:** 1 Department of Biology, College of Science, Sultan Qaboos University, P. O. Box 36, Muscat, Oman; 2 Department of Genetics, College of Medicine, Sultan Qaboos University, P. O. Box 35, Muscat, Oman; 3 Department of Life Sciences, Hogeschool van Arnhem en Nijmegen, Kapittelweg 33, 6525 Nijmegen, EN Netherlands

**Keywords:** Breast cancer, NRP-1, ABCG2, TNC, Integrin beta 3, Chemoresistance, Adriamycin, Cyclophosphamide

## Abstract

**Background:**

Neuropilin-1 (NRP-1), a non-tyrosine kinase glycoprotein receptor, is associated with poor prognosis breast cancer, however transcriptomic changes triggered by NRP-1 overexpression and its association with chemoresistance in breast cancer have not yet been explored.

**Methods:**

BT-474 NRP-1 variant cells were generated by stable overexpression of NRP-1 in the BT-474 breast cancer cell line. RNA sequencing and qRT-PCR were conducted to identify differentially expressed genes. The role of an upregulated oncogene, *Tenascin C (TNC)* and its associated pathway was investigated by siRNA-mediated knockdown. Resistant variants of the control and BT-474 NRP-1 cells were generated by sequential treatment with four cycles of Adriamycin/Cyclophosphamide (4xAC) followed by four cycles of Paclitaxel (4xAC + 4xPAC).

**Results:**

NRP-1 overexpression increased cellular tumorigenic behavior. RNA sequencing identified upregulation of an oncogene, *Tenascin-C* (*TNC)* and downregulation of several tumor suppressors in BT-474 NRP-1 cells. Additionally, protein analysis indicated activation of the TNC-associated integrin β3 (ITGB3) pathway via focal adhesion kinase (FAK), Akt (Ser473) and nuclear factor kappa B (NF-kB) p65. siRNA-mediated *TNC* knockdown ablated the migratory capacity of BT-474 NRP-1 cells and inactivated FAK/Akt473 signaling. NRP-1 overexpressing cells downregulated breast cancer resistance protein (BCRP/ABCG2). Consequently, sequential treatment with Adriamycin/Cyclophosphamide (AC) cytotoxic drugs to generate resistant cells indicated that BT-474 NRP-1 cells increased sensitivity to treatment by inactivating NRP-1/ITGB3/FAK/Akt/NF-kB p65 signaling compared to wild-type BT-474 resistant cells.

**Conclusions:**

We thus report a novel mechanism correlating high baseline NRP-1 with upregulated *TNC*/ITGB3 signaling, but decreased ABCG2 expression, which sensitizes BT-474 NRP-1 cells to Adriamycin/Cyclophosphamide. The study emphasizes on the targetability of the NRP-1/ITGB3 axis and its potential as a predictive biomarker for chemotherapy response.

**Electronic supplementary material:**

The online version of this article (10.1186/s12885-018-4446-y) contains supplementary material, which is available to authorized users.

## Background

Breast cancer remains the most common cancer and the second most common cause of cancer-related deaths in women [1]. The role of Neuropilin-1 (NRP-1), a multifunctional transmembrane protein that interacts with a multitude of signaling receptors, in breast cancer pathogenesis has been extensively investigated. Previously, we showed that NRP-1 is overexpressed in malignant subtypes of epithelial ovarian cancer and was positively correlated with epithelial to mesenchymal transition (EMT) markers [2]. In addition, we detected high NRP-1 expression in breast cancer MDA-MB-231 parental and metastatic variant cells [3]. Most recently, we reported upregulated plasma and tumor tissue expression of NRP-1 in breast cancer cases with advanced nodal and metastatic disease, and particularly in triple negative breast cancer compared to other molecular subtypes [4]. Overexpression of NRP-1 has also been observed in several other cancer types and is associated with tumor progression and poor patient prognosis [5]. A monoclonal antibody against NRP-1 has been tested in phase I clinical trials and shown to enhance bevacizumab-mediated vascular endothelial growth factor (VEGF) pathway blockade [6, 7]. The crosstalk between integrin αvβ3 and NRP-1 in endothelial cells limits the latter in contributing to VEGF-induced angiogenesis [8]. It has been shown that the suppression of Integrin β3 (ITBG3) in endothelial cells activates cell migration pathway through NRP-1 mobilization away from mature focal adhesions following VEGF-stimulation [9]. In addition, integrin αvβ3 was shown to control the metastatic ability of breast cancer cells to the brain in a mouse model [10]. Integrin αvβ3 binds to many extra cellular matrix proteins and enhances proliferation and migration through the phosphorylation of the downstream signaling of the focal adhesion kinase pathway (FAK) [11]. Previously we showed the exclusive expression of ITGB3 in brain metastasized MDA-MB-231 cells [3]. However, the effect of NRP-1 overexpression on the ITGB3 signaling pathway and its role in chemoresistance in breast cancer has not yet been investigated. Furthermore, the role of NRP-1 in oncogenesis has been extensively investigated, in an era of advancement in omics technology there is a lack of studies dissecting the global changes elicited by NRP-1 modulation. This is of importance to identify novel pathways associated with NRP-1 function with the purpose of dual targeting in breast cancer patients to increase treatment efficiency. An additional hindrance in breast cancer treatment is the high mortality associated with chemoresistance [12]. Thus, dissecting mechanisms underlying the ability of tumor cells to develop resistance to cytotoxic drugs is of high priority in the race towards targeted and personalized therapeutics and the development of biomarkers to predict patient prognosis and drug response. Therefore, the main objectives of this study were to understand novel functional mechanism by which NRP-1 enhances breast cancer progression and to investigate the relationship of NRP-1 with acquired chemoresistance.

## Methods

### Cell culture

Human breast cancer cell lines BT-474 (300131), MCF7 (300273) and MDA-MB-231 (300275) were purchased from Cell Lines Service CLS (Germany), which authenticates cell lines using the STR DNA analysis method. BT-474 cells were grown in RPMI-160 (Gibco, USA) supplemented with 10% FBS, 50 μg/ml gentamicin and sodium pyruvate (Gibco, USA). All cells were maintained at 37 °C in 5% CO_2_ incubators.

### Stable transfection of NRP-1

The NRP-1 cDNA (GenBank accession number BX510902.1) cloned in pDONR221 plasmid (Cat. HsCD00295948) was purchased from DNASU Plasmid Repository (USA). The cDNA fragment (2750 bp coding for the amino acids from 1 to 917 of the protein) was amplified using the following primers: NRP-1 F: 5′- GGAATTCTATGGAGAGGGGGCTGC-3′ and NRP-1R: 5′- CGGGATCCTGTGTATTCAGTTTGTCTTT-3′, purified using the GeneJET Gel Extraction Kit (Thermo Fisher Scientific, Lithuania), digested with *Eco*R-1 and *Bam*H-1 and subcloned in frame in the pPTuner IRES2 expression plasmid (Takara Bio, USA). The cloned vector was electroporated using the Gene Pulser electroporation system (Bio-Rad, USA) into *DH10B* competent *E. coli* cells and the positive clones were selected on LB Kanamycin plates (100 mg/ml) and confirmed by restriction enzyme analysis and DNA sequencing (Macrogen Inc., Korea). The resulting vector was stably transfected into the BT-474 cell line using Lipofectamine-2000 and positive transfected clones were isolated by the colony disk isolation method and selected using 600 μg/ml of Geneticin G418 antibiotic (Gibco, USA). The transfected cells were designated as BT-474 NRP-1. The empty plasmid was also transfected into BT-474 cells and used as a negative vector control.

### Generation of chemo-resistant lines

Chemoresistant BT-474 and BT-474 NRP-1 variant cell lines were generated in a similar protocol to that described previously [13]. Briefly, the cells were treated with four cycles of a combination of 0.5 uM of Doxorubicin (Brand name Adriamycin, Pharmacia, Italy) + 300 nM Cyclophosphamide (Brand name Cytoxan, Baxter, Germany) (cells will be referred to as 4xAC) followed by four cycles of 20 nM Paclitaxel (Brand name Taxol, EBEWE Pharma, Austria) (cells will be referred to as 4xAC + 4xPAC). Each cycle was for a duration of 72 h followed by a recovery period until confluency was achieved prior to commencement of the next cycle. Protein lysate and RNA was extracted from the 4xAC and 4xAC + 4xPAC resistant cell lines and stored at − 80 °C.

### Western blotting

Cells were lysed in 1× lysis buffer (Cell Signaling Technology, USA) supplemented with phenylmethylsulfonyl fluoride (PMSF) protease inhibitor (Sigma, Germany). Western blotting was performed according to a standard protocol as described in [3]. The primary antibodies used are listed in Additional file 1: Table S1. HRP-linked secondary rabbit/mouse antibody was utilized to detect the chemiluminescence signal using Clarity ECL (Bio-Rad) and visualized using the ChemiDoc Touch Imaging System (Bio-Rad). Images were acquired and processed with the Image Lab software Version 5.2.1 (Bio-Rad).

### Quantitative real-time PCR

RNA extraction and qRT-PCR were performed according to standard protocols as described earlier [4]. Primers were designed using the Primer Express software (Applied Biosystems, USA) and are listed in Additional file 1: Table S2.

### Proliferation assay

The AlamarBlue® (GeneCopoeia, USA) proliferation assay was carried out according to the manufacturer’s instructions using 80,000 cells/well.

### Invasion assay

The invasive capacity of the cells was determined using the CultreCoat 96 Well Medium BME cell invasion assay (Trevigen, USA) according to the manufacturer’s instructions using 25,000 cells/insert. The fluorescence was measured at 485 nm excitation and 520 nm emission using an Epoch Microplate Spectrophotometer (BioTek, USA) and the Gen5 software version 2.07.

### Clonogenic assay

In this assay, 10^4^ cells/well were seeded in six-well plates and maintained in complete growth media for 14 days after which the cells were washed and stained with crystal violet (5% Bromophenol Blue + 25% methanol) for 20 mins. The excess stain was washed off with distilled water and the stained colonies were counted manually.

### Spheroid formation

Here, 10^4^ cells/well were seeded in 96-well plates coated with 5% agarose to reduce surface binding, and maintained for seven days in complete growth media. Microscopic images were taken daily using the Axio Vert.A1 microscope, Axiocam ERc 5 s camera (Zeiss, Germany) and AxioVision software version 4.9.

### Wound healing assay/migration assay

One million cells were seeded in 25 cm^2^ flasks (Thermo Fisher Scientific, USA) and cultured until 90% confluency. A wound was generated in the monolayer with a sterile glass tip. The ability of the cells to migrate towards each other and close the gap generated was assessed by microscopic imaging.

### Immunofluorescence microscopy

Here, 10^6^ cells were seeded on a sterile positively charged slide, fixed with 4% Paraformaldehyde, permeabilized using 0.05% Triton X-100, followed by blocking with 5% goat serum and overnight incubation with a mixture of primary anti-rabbit NRP-1 antibody (1:100) (Abcam, UK) and anti-mouse TNC antibody (1:200) (Santa Cruz, USA) diluted in PBS. Anti-rabbit IgG Fab2 Alexa Fluor 555 and anti-mouse IgG Fab2 Alexa Fluor 488 secondary antibody (Cell Signaling Technology) were used at a 1:400 dilution in PBS. Cells were counterstained with DAPI (1:250). Images were captured using a Nikon H600L fluorescent microscope (Japan) and NIS Elements software version 4.40.

### RNA sequencing

Next generation sequencing using the Illumina HiSeq 2500 sequencing system was performed at CD Genomics (USA) for two replicates each of the control BT-474 and BT474-NRP1 cells. A total of 22,655,647 clean reads from 22,914,684 raw sequencing reads were generated upon sequencing. The count per million (CPM) method was utilized for filtering low counts/noise by NOISeq. The clean reads were mapped to the reference genome using the HISAT [14]/ Bowtie2 tool [15]. The fragments per kilobase of transcript per million mapped reads (FPKM) method was utilized to calculate the expression levels. A false detection rate (FDR) ≤ 0.001 and the absolute value of Log_2_ ratio ≥ 2 were used as the default threshold to identify significant DEGs. DEGs were classified based on their Gene Ontology (GO) functional terms and KEGG pathway enrichment analysis using the Blast2GO software. RNA sequencing results were validated using quantitative real-time PCR.

### siRNA- mediated transient knockdown

In this case, 2 × 10^5^ BT-474 NRP-1 cells/well were seeded in a six-well plate in complete growth medium until 70% confluency. Cells were treated with 80 pmol of a pool of three human TNC-targeted siRNA (Santa Cruz) or control siRNA in transfection reagent and serum-free siRNA transfection medium (Santa Cruz) according to the manufacturer’s instructions.

### Statistical analysis

An independent samples t-test was utilized to identify statistical differences between the BT-474 and BT-474/NRP-1 cell lines. Analysis of variance (ANOVA) with the Tukey post hoc test was utilized to determine statistical differences between the resistant cell lines. Statistical analysis was based on three independent replicates. A *p*-value < 0.05 was regarded as the threshold for statistical significance.

## Results

### Recombinant NRP-1 overexpression increased the tumorigenic ability of BT-474

**Fig. 1:**
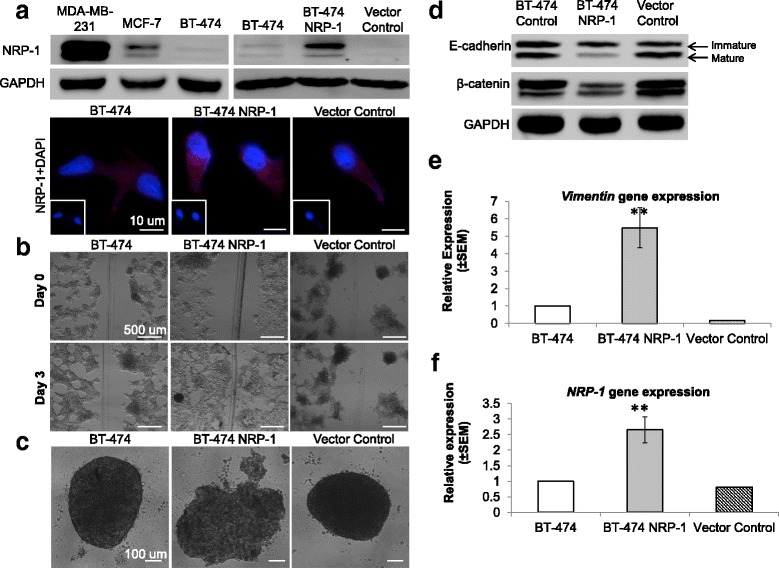
NRP-1 overexpression in BT-474 cell line triggers tumorigenic features. **a.** Comparison of NRP-1 expression in three breast cancer cell lines MDA-MB-231, MCF-7 and BT-474. NRP-1 was stably overexpressed in the BT-474 cell line and confirmed at the level of protein expression by western blotting and immunofluorescence staining (40× magnification, scale bar 10 μm). **b.** Wound healing assay images (5× magnification, scale bar 500 μm) taken on day 0 and day 3 indicate increased migration in BT-474 NRP-1 cells compared to control cells 3 days post wound generation. **c.** NRP-1 overexpression reduced spheroid formation (20× magnification, scale bar 100 μm). **d.** Western blotting indicated decreased expression of mature form of E-cadherin (lower band) and β-catenin along with **e,** significantly increased *vimentin* and **f,** *NRP-1* gene expression. Gene expression is relative to the control BT-474 and normalized to *β-Actin* and *GUSB* reference genes expression. The graph represents the mean ± SEM of three independent experiments. Statistical analysis using independent samples t-test, *p*-value < 0.05 considered as statistically significant ** *p* < 0.01

In order to understand the functional role of NRP-1 in breast cancer progression, NRP-1 was stably overexpressed in the BT-474 cell line, which has very low baseline levels of NRP-1 compared to other breast cancer cell lines such as MDA-MB-231 and MCF-7 (Fig. 1a). NRP-1 overexpression in the transfected BT-474 NRP-1 cells was confirmed at protein level by western blotting and immunofluorescence microscopy (Fig. 1a). BT-474 NRP-1 cells displayed significantly increased migration compared to control cells three days post generation of the wound (Fig. 1b). Moreover, NRP-1 overexpression decreased the ability of the cells to form spheroids. Control BT-474 cells formed compact dense spheroids, but BT-474 NRP-1 cells either did not form spheroids or formed less dense clumps that did not adhere to form distinct spheroids (Fig. 1c). NRP-1 overexpression decreased the levels of proteins related to adhesion and migration as shown in a decrease in the cleaved active form of E-cadherin (lower band) and β-catenin (Fig. 1d), and a significant increase in gene expression of *vimentin* (Fig. 1e) similar to *NRP-1* gene expression (Fig. 1f). NRP-1 overexpression increased the clonogenic ability of the cells as observed from the increased number of colonies in the BT-474 NRP-1 cells compared to control BT-474 (Additional file 2: Figure S1A). In addition, NRP-1 overexpression significantly increased the proliferation of the BT-474 NRP-1 cells (Additional file 2: Figure S1B). However, there was a non-significant increasing trend in the invasion ability of the BT-474 NRP-1 variant cells compared to the control BT-474 cells (Additional file 2: Figure S1C).

### RNA-Seq identified novel genes associated with NRP-1 overexpression

**Fig. 2:**
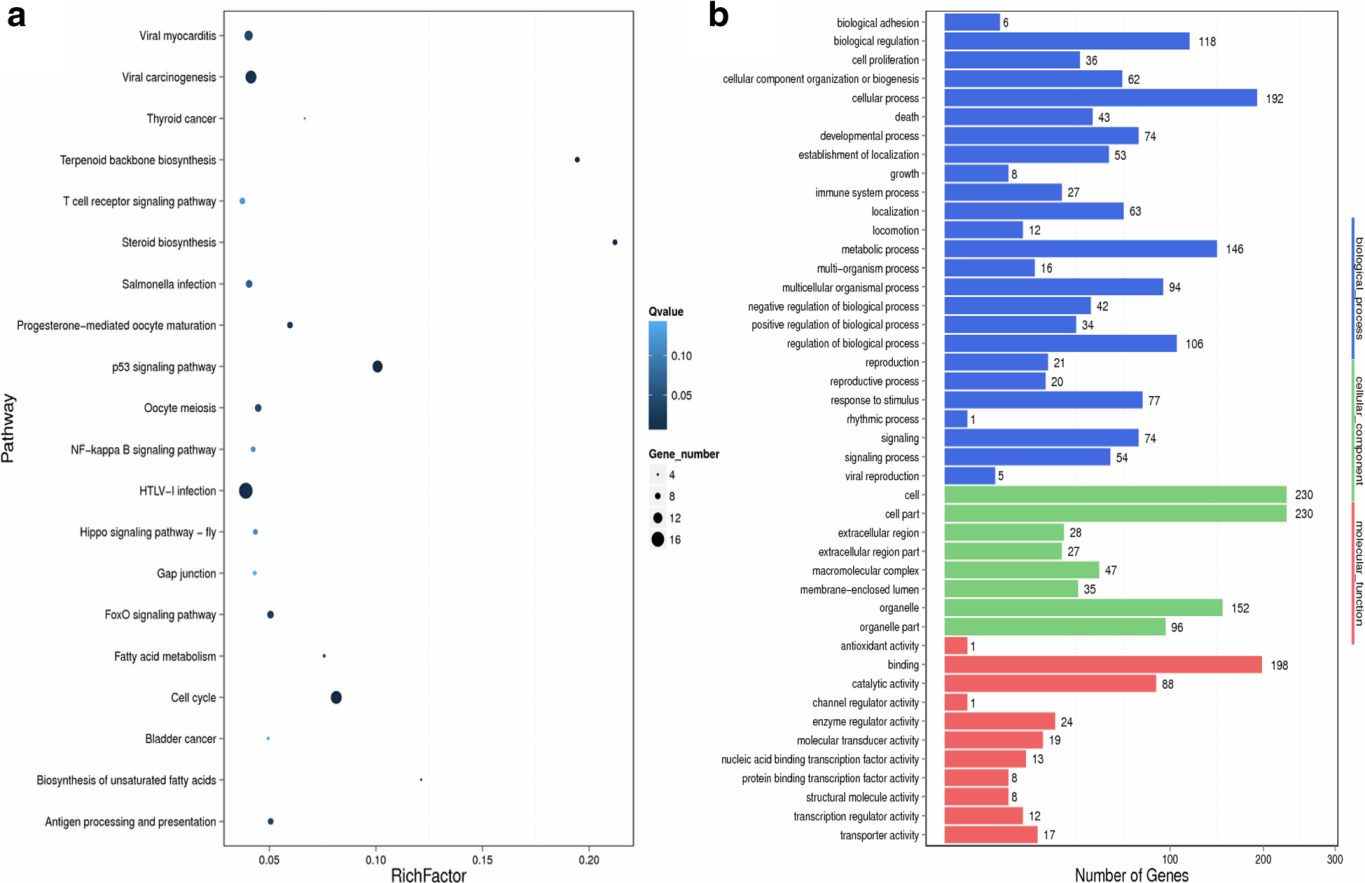
Enrichment of cancer-related pathways from transcriptome-wide analysis. **a.** Pathway enrichment and **b.** Gene Ontology analysis of differentially expressed genes (DEGs) between the BT-474 and BT-474 NRP-1 cells from RNA sequencing based on a Log_2_ ratio cutoff ≥2 and concurrence in 2 replicates

**Fig. 3:**
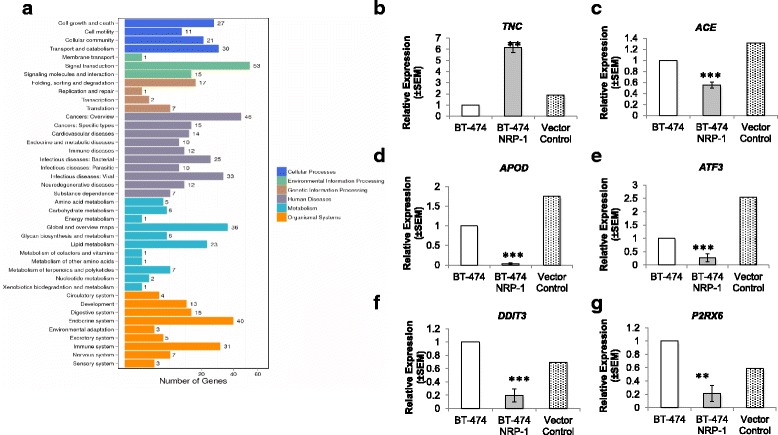
Confirmation of DEGs from transcriptome analysis. **a.** KEGG pathway analysis of DEGs between BT-474 and BT-474 NRP-1 cells identified from RNA sequencing. Real-time qPCR was implemented on shortlisted DEGs obtained from RNA sequencing to confirm their differential expression. BT-474 NRP-1 cells significantly upregulated **b.** *TNC* expression and downregulated **c,** ACE, **d,** *APOD*, **e,** *ATF3*, **f,** *DDIT3* and **g,** *P2RX6* expression. Cells transfected with the empty plasmid indicated similar expression to the BT-474 cells. Graphs represent the mean ± SEM of 3 independent experiments. Statistical analysis using independent samples t-test, *p* value < 0.05 considered as statistically significant ** *p* < 0.01, ***p < 0.001

Since NRP-1 overexpression triggered several dynamic phenotypic changes, we further sought to determine the resulting global transcriptomic variations in the BT-474 NRP-1 variant cells. 23 upregulated and 61 downregulated genes were identified in the NRP-1 overexpressing cells in comparison to wild-type BT-474 cells (Additional file 1: Table S3 and S4). Pathway analysis indicated an enrichment of genes related to viral carcinogenesis, p53 signalling pathway and the cell cycle in the BT-474 NRP-1 cells (Fig. 2a) and Binding was the most enriched gene ontology GO term related to molecular function (Fig. 2b). Kyoto encyclopedia of genes and genomes (KEGG) classification also indicated an enrichment of genes related to signal transduction and cancers (Fig. 3a). Among the DEGs, 11 upregulated and 11 downregulated genes were shortlisted based on their known roles in cancer and/or EMT for confirmation with real-time qPCR (Additional file 1: Table S5). Consistent with observations from RNA-Seq, Tenascin C (*TNC)* was significantly upregulated in the BT-474 NRP-1 cells in 4 independent replicas (Fig. 3b). In addition, Angiotensin Converting Enzyme (*ACE)* (Fig. 3c), Apolipoprotein D (*APOD)* (Fig. 3d), Activating Transcription Factor 3 (*ATF3)* (Fig. 3e), DNA Damage induced transcript 3 (*DDIT3)* (Fig. 3f) and P2X purigenic receptor 6 (*P2RX6)* (Fig. 3g) genes were significantly downregulated in the BT-474 NRP-1 cells. The vector control cells indicated similar expression of these genes to the wild-type BT-474 cells (Fig. 3b-f).

### *Tenascin C* contributes to the increased migratory capacity of BT-474 NRP-1 cells

**Fig. 4:**
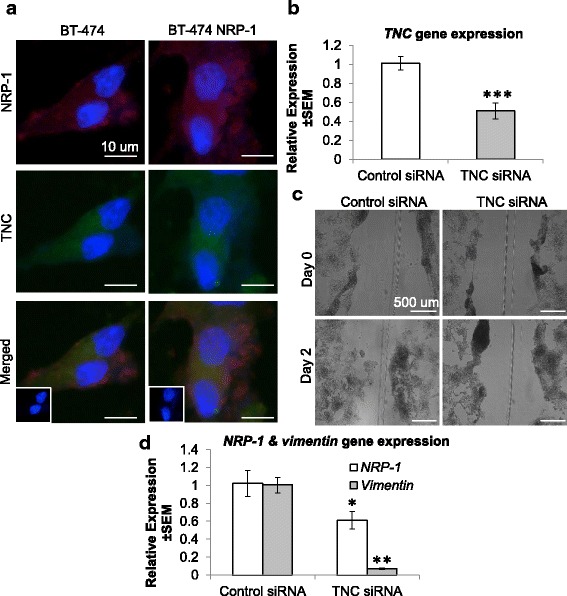
Tenascin C contributes to NRP-1 associated migration. **a.** Dual immunofluorescence staining (40× magnification scale bar 10 μm) of NRP-1 and TNC on BT-474 and BT-474 NRP-1 cells indicates their colocalization in the cytoplasm. Treatment of BT-474 NRP-1 cells with TNC targeted siRNA molecules, **b**, reduced *TNC* gene expression, **c**, reduced migratory capacity and **d**, downregulated *NRP-1* and *vimentin* expression. (TNC protein was not detected on western blot due to the lack of specific antibody for this application.) The gene expression fold change was measured by comparing the basal levels detected in the empty plasmid transfected BT-474 or in the case of the siRNA experiment, to the control siRNA treated BT-474 and normalized to *β-Actin* and *GUSB* reference gene expression. Wound healing assay images (panel d, 5× magnification, scale bar 500 μm) taken on day 0 and day 2 after siRNA transfection. Graphs represent the mean ± SEM of three independent experiments. Statistical analysis using independent samples t-test, p-value < 0.05 considered as statistically significant. *p < 0.05, ** *p* < 0.01, *** *p* < 0.001

A candidate DEG, *TNC,* confirmed to be significantly upregulated by RNA-Seq and qPCR, was selected based on its known oncogenic function. The upregulation of *TNC* gene expression was confirmed at the protein level where BT-474 NRP-1 cells expressed a high cytoplasmic granular expression of TNC (Fig. 4a). Moreover, TNC protein expression colocalized with NRP-1 in the cytoplasm but not on the cell membrane (Fig. 4a). Knockdown of *TNC* expression using a pool of siRNA resulted in a 50% downregulation in gene expression (Fig. 4b). *TNC* downregulation significantly reduced the migratory capacity of the BT-474 NRP-1 cells (Fig. 4c), which was supported by a concomitant and significant decrease in *NRP-1* and *vimentin* expression (Fig. 4d).

### BT-474 NRP-1 cells activate TNC-associated ITGB3/FAK/Akt/NF-kB and TNFR2 signaling

**Fig. 5:**
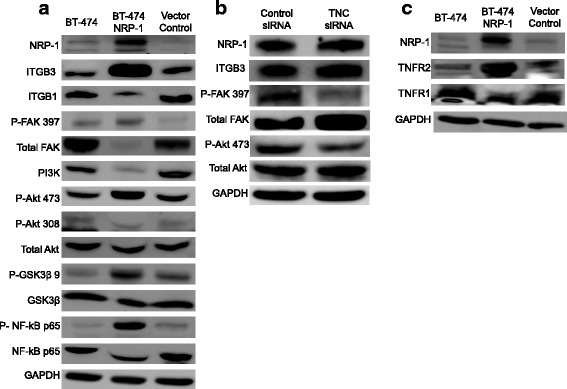
NRP-1 overexpression activates integrin β3 and TNFR2 pathways. Representative western blot images of protein lysates from untransfected BT-474, BT-474 NRP-1 and empty vector control cells blotted with indicated antibodies involved in **a.** Integrin signaling, and downstream signaling targets FAK, Akt, GSK3-β and NF-kB **b.** siRNA-mediated *TNC* downregulation decreased phosphorylation of FAK and Akt-473. **c.** Blots show levels of tumor necrosis factor receptors (TNFRs). GAPDH protein expression is indicated as a loading control. (The prefix P beside the antibody names indicates the phosphorylated form)

Since TNC is known to signal via integrins on the cell surface, the expression of the integrin family was quantified. NRP-1 overexpression was found to significantly upregulate integrin β3 (ITGB3) (Fig. 5a); however, it downregulated integrin β1 (ITGB1) (Fig. 5a) and did not alter the expression of integrin β4 and β5 (data not shown). The expression of integrins α5, alpha-V and alpha4 could be detected neither in the control nor in NRP-1 overexpressing BT-474 cells. NRP-1 overexpression increased the phosphorylation (Tyr397) of focal adhesion kinase (FAK), a known integrin effector molecule (Fig. 5a). Although a decrease in the phosphatidyl inositol-3 kinase (PI3K) 110α catalytic subunit was observed, Akt, the downstream target of FAK, was activated in BT-474 NRP-1 cells with increased phosphorylation at site Ser473 but not site Ser308 (Fig. 5a). Akt-473 phosphorylation was further associated with a concomitant increase in glycogen synthase kinase 3-beta (GSK3β) phosphorylation at Ser9 (Fig. 5a) and activation of the downstream nuclear factor kappa B (NF-kB) pathway as observed from the upregulation of phosphorylated NF-kB p65 (Fig. 5a). siRNA-mediated *TNC* knockdown in BT-474 NRP-1 cells reversed FAK and Akt activation by downregulating the phosphorylation of FAK at Tyr397 and Akt at Ser473 but did not affect the expression of NRP-1 (protein) or ITGB3 as shown in the representative western blots (Fig. 5b). TNC protein band was not detected on western blot due to the lack of proper antibody for this application.

As the Akt-473 pathway was observed to be upregulated in the BT-474 NRP-1 cells, the expression of additional upstream regulators of Akt signaling were analyzed. We found that BT-474 NRP-1 cells also displayed a significant upregulation of tumor necrosis factor receptor 2 (TNFR2) and downregulation of TNFR1 expression (Fig. 5c).

### BT-474 NRP-1 cells have increased sensitivity to Adriamycin/cyclophosphamide exposure

**Fig. 6:**
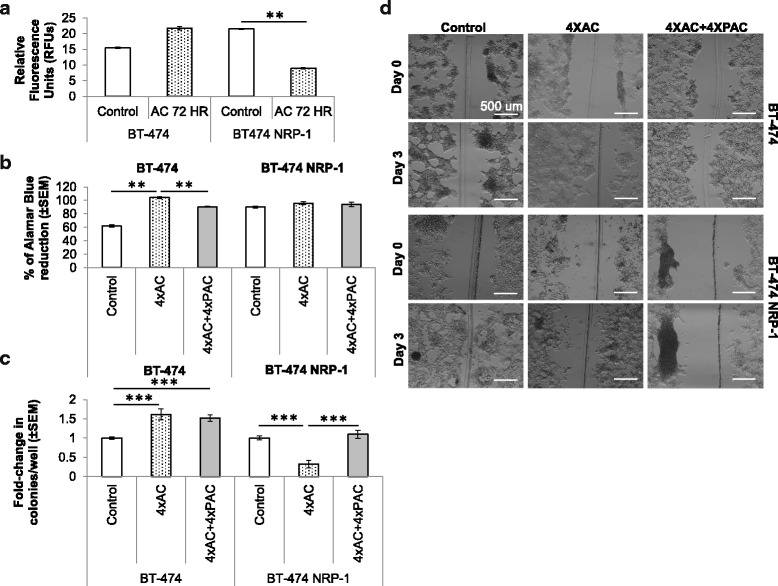
Overexpression of NRP-1 caused differential cellular responses to cytotoxic treatment. **a.** Treatment with 0.5 uM Adriamycin + 300 nM Cyclophosphamide (AC) for 72 h significantly decreased the invasive capacity of the BT-474 NRP-1 cells but indicated an increasing trend in the invasion ability of BT-474 cells. Resistant BT-474 and BT-474 NRP-1 cells were generated by long-term treatment (eight months) with either four cycles of AC alone (4xAC) or a combination of four cycles of AC and four cycles of 20 nM Paclitaxel (4xAC + 4xPAC), and cellular responses were assessed based on their **b**, proliferation and **c**, clonogenic ability, whereby BT-474 4xAC cells indicated significantly increased proliferation and clonogenic capacity compared to BT-474 NRP-1 4xAC cells. **d.** Wound healing assay images (5× magnification, scale bar 500 μm) to determine the migratory capacity of chemoresistant cells 72 h post generation of the wound indicated decreased migration in BT-474 NRP-1 overexpressing 4xAC and 4xAC + 4xPAC resistant cells compared to BT-474 resistant cells. Fold change in colonies/well (panel **c**) is compared to the respective untreated BT-474 or BT-474 NRP-1 cells. Graphs represent the mean ± SEM of three independent experiments. Statistical analysis using ANOVA and Tukey post hoc test, *p*-value < 0.05 considered as statistically significant. ** *p* < 0.01, ***p < 0.001

To determine the role of NRP-1 in cellular response to cytotoxics, BT-474 control and BT-474 NRP-1 variant cells were treated with a combination of Adriamycin and Cyclophosphamide (AC) for 72 h, and their capacity to invade was assessed. Despite the more tumorigenic behavior of the BT-474 NRP-1 cells, they indicated increased sensitivity to the AC treatment as observed from the significantly reduced invasive capacity whereas the control BT-474 cells indicated a trend to increased invasion (Fig. 6a). To further understand the effect of baseline NRP-1 overexpression in increasing chemosensitivity, cells chemoresistant to AC and AC + PAC were generated as described in the methods section, and their properties analyzed. In terms of the resistant variants of the wild-type BT-474 cells, the ability of the BT-474 4xAC cells to proliferate was significantly increased compared to untreated control BT-474 cells (Fig. 6b), however the addition of Paclitaxel in the 4xAC + 4xPAC resistant cells reversed the action of 4xAC by decreasing the proliferative capacity of the cells to a level similar to untreated control cells (Fig. 6b). On the other hand, in the BT-474 NRP-1 cells, the 4xAC and 4xAC + 4xPAC resistant variants did not indicate alterations in proliferation compared to untreated cells (Fig. 6b). In terms of colony formation/clonogenic ability in the wild-type BT-474 resistant cells, the BT-474 4xAC and 4xAC + 4xPAC cells formed significantly more colonies compared to untreated control cells (Fig. 6c). On the other hand, in the NRP-1 overexpressing cells, BT-474 NRP-1 4xAC cells showed a significantly reduced ability to form colonies, which was increased in BT-474 NRP-1 4xAC + 4xPAC resistant cells to a level similar to untreated cells (Fig. 6c).

**Fig. 7:**
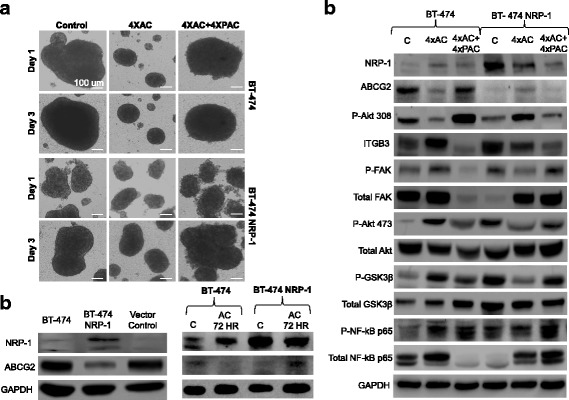
Differential spheroid formation capacity and molecular profiles in NRP-1 overexpressing chemoresistant cells. **a.** Spheroid formation (20× magnification, scale bar 100 μm) assessed after 24 and 72 h in BT-474 and BT-474 NRP-1 resistant cells indicated multiple smaller spheroids in 4xAC resistant cells but similar phenotypes between the 4xAC + 4xPAC and their respective untreated controls. **b.** Representative western blots to indicate NRP-1 overexpression inversely correlates with a downregulation of breast cancer resistant protein (BCRP/ABCG2) in untreated cells and on short term AC treatment for 72 h. GAPDH protein expression is indicated as a loading control. **c.** Representative images from western blot analysis of BT-474 and BT-474 NRP-1 untreated, 4xAC and 4xPAC cell lysates blotted with the indicated antibodies. NRP-1 and integrin β3 pathway molecules indicate a similar expression profile of significantly upregulated expression in BT-474 4xAC cells but a downregulation in BT-474 NRP-1 4xAC cells compared to their respective untreated controls. Images are representative of independent experiments with comparable outcomes. GAPDH protein expression is indicated as a loading control. (The prefix P beside the antibody names indicates the phosphorylated form)

The ability of the resistant cells to migrate and form spheroids was also assessed. In wild-type BT-474 resistant cells, the wound-healing assay indicated increased migration of the BT-474 4xAC and 4xAC + 4xPAC cells compared to the untreated control cells (Fig. 6d). However, in the BT-474 NRP-1 resistant cells, both 4xAC and 4xAC + 4xPAC cells displayed decreased migration, with the BT-474 NRP-1 4xAC + 4xPAC cells displaying minimal migration compared to the untreated cells (Fig. 6d). In the spheroid formation assay, wild-type BT-474 cells had the ability to form distinct, dense spheroids within 24 h of cell seeding, whereas 4xAC resistant cells formed multiple smaller spheroids that remained separate even 72 h post seeding (Fig. 7a). Treatment with 4xAC + 4xPAC reverted the spheroid phenotype to one similar to untreated BT-474 cells (Fig. 7a). On the other hand, the variant BT-474 NRP-1 untreated and 4xAC and 4xAC + 4xPAC resistant cells displayed multiple loose spheroids 24 h post cell seeding (Fig. 7a). After 72 h, while the untreated variant BT-474 NRP-1 spheroids had merged together to form a single large spheroid, the 4xAC spheroids had merged to an extent but still displayed multiple spheroids (Fig. 7a). The BT-474 NRP-1 4xAC + 4xPAC spheroids merged to form a single large, albeit loose, spheroid similar to their untreated counterpart (Fig. 7a).

### NRP-1 is inversely related to BCRP/ABCG2 expression

Interestingly, the expression of breast cancer resistance protein (BCRP/ABCG2) was also found to be downregulated in BT-474 NRP-1 variant cells compared to control BT-474 and increased on short-term AC treatment, inversely to NRP-1 (Fig. 7b). Moreover, the expression of ABCG2 and Phospho-Akt Ser308 indicated an opposing trend to NRP-1 expression in the resistant cells also (Fig. 7c). In the control BT-474 cell line, both ABCG2 and Phospho-Akt Ser308 decreased in 4xAC resistant cells and increased to a level similar to control levels in the resistant 4xAC + 4xPAC cells (Fig. 7c). The BT-474 NRP-1 cells, which express significantly lower levels of ABCG2 and Phospho-Akt 308 compared to control BT-474 cells, showed an upregulation in cells resistant to 4xAC and a decrease similar to untreated cells in the 4xAC + 4xPAC resistant cells (Fig. 7c).

### NRP-1 and ITGB3 signaling are co-expressed and differentially regulated in BT-474 NRP-1 chemoresistant cells

To identify the molecular changes regulating the altered chemosensitivity in the BT-474 NRP-1 cells, the expression of NRP-1 and the associated activation of the integrin signaling pathway was determined. In terms of the wild-type BT-474, resistance to 4xAC was associated with an upregulation of NRP-1, ITGB3 and its downstream mediators Phospho-FAK Tyr397, Phospho-Akt Ser473 and Phospho-GSK3β, as well as the downstream transcriptional factor Phospho-NF-kB p65, compared to untreated control BT-474 cells (Fig. 7c). BT-474 resistant to 4xAC + 4xPAC reversed the effect of 4xAC, by downregulating the expression of NRP-1, ITGB3, Phospho-FAK Tyr397, Phospho-Akt Ser473 and Phospho-GSK3β (Fig. 7c). The level of Phospho-NF-kB p65 still remained upregulated similar to the expression in 4xAC cells (Fig. 7c).

In contrast, the NRP-1 overexpressing variant BT-474 NRP-1 4xAC cells displayed a downregulation of NRP-1, ITGB3, Phospho-FAK Tyr397, Phospho-Akt Ser473, Phospho-GSK3β and Phospho-NF-kB p65 compared to untreated cells (Fig. 7c). While the expression of NRP-1 and ITGB3 was further downregulated in the BT-474 NRP-1 cells treated with 4xAC + 4xPAC, the addition of Paclitaxel reversed the effect of 4xAC by upregulation of Phospho-FAK Tyr397, Phospho-Akt Ser473, Phospho-GSK3β and Phospho-NF-kB p65 (Fig. 7c).

## Discussion

The role of NRP-1 in triggering tumorigenic behavior has been indicated in several cancer types, including breast cancer [16, 17]. Moreover, depending on the ligand binding to NRP-1, it can have a dual role in different types of cells [18, 19]. Despite the numerous roles of NRP-1, global transcriptomic effects elicited by NRP-1 in cancer cells have not been previously explored and more importantly, its role in the response to chemotherapy in breast cancer has not been investigated either. In this functional study, we overexpressed NRP-1 and conducted protein analysis and total RNA sequencing to understand global changes triggered by recombinant NRP-1 overexpression. NRP-1 is coded by at least 5 splice and genetic variants [20]. In this project we overexpressed the largest NRP-1 variant which has changes in the last six amino acids (STYSEA versus DPPLSL) at the C-terminus. As the BT-474 NRP-1 cells exhibit typical cellular tumourigenic phenotypes as described in this study, these conserved amino acids may not be crucial for NRP-1 function in this context. We report several genes that have not previously been associated with NRP-1 overexpression, which include oncogenes, tumor suppressors and genes involved in cancer-related pathways and protein binding function. We describe a novel association between NRP-1 and *TNC* expression, an extracellular matrix (ECM) glycoprotein molecule that induces EMT, migration, proliferation and immune modulations in cancer [11, 21]. TNC triggers the cytoplasmic translocation of E-cadherin and β-catenin by Src-mediated FAK signaling in combination with ανβ1 and ανβ6 integrins, thus leading to enhanced migratory behavior [22]. TNC knockdown in invasive breast cancer cells was shown to reduce proliferation and invasion [23]. The common essentiality of both NRP-1 and TNC in neuronal development provides evidence for the presence of a close association between these molecules [24, 25]. Based on our observations, we report the contribution of *TNC* to NRP-1-triggered cell migration and activation of the FAK/Akt signaling pathway. NRP-1 and *TNC* upregulation was also associated with significantly increased *vimentin* expression and a downregulation in EMT and the tumor suppressor molecules E-cadherin (mature form) and β-catenin, that contribute to the increased clonogenic ability, migration and loss of cell-cell adhesion in the variant cells [17].

Integrins are cellular receptors that interact with the ECM and in turn activate downstream intracellular signaling pathways [26]. The dependence of NRP-1 on integrin signaling was not previously reported in breast cancer. In the BT-474 NRP-1 model, ITGB1 was downregulated and accompanied by a significant upregulation in ITGB3 expression. The loss of ITGB1 provides further evidence for the molecular mechanism of the lack of spheroid formation in the BT-474 NRP-1 cells [27]. ITGB3 expression has been linked with tumor aggression [28] and metastasis to the bone and brain in breast cancer [3] by a mechanism involving FAK and Akt signaling [29]. Our study in the BT-474 breast cancer model provides further insight into this pathway by highlighting the role of NRP-1 in triggering ITGB3/FAK/Akt-473 in a TNC-dependent pathway to trigger cell migration. Interaction studies to investigate the physical association between NRP-1, ITGB3 and TNC were limited by the lack of availability of a suitable antibody for TNC for immunoprecipitation and western blotting.

In addition, the BT-474 NRP-1 clones appear to be signaling via multiple pathways including the activation of the pro-survival TNFR2 pathway and downregulation of the apoptotic TNFR1 pathway [30], additionally explaining the increased proliferation and clonogenic ability of NRP-1 overexpressing cells. Like ITGB3, TNFR2 is also known to activate Akt phosphorylation and hence, promote cellular proliferation/survival [31]. The phosphorylation of Akt at site Ser473 in our model may be directly dependent on FAK but independent of PI3K which mainly phosphorylates Akt at Ser-308 [32], both of which were downregulated in the BT-474 NRP-1 cells. The activation of Akt was also accompanied by the phosphorylation and inactivation of GSK3β in the NRP-1 overexpressing BT-474 variant cells. Although the role of GSK3β is controversial, it is known to act as a tumor suppressor by regulating cell cycle proteins [33]. Thus, Akt-mediated inhibition of GSK3β may provide an additional mechanism by which NRP-1 exerts its pro-survival function.

**Fig. 8:**
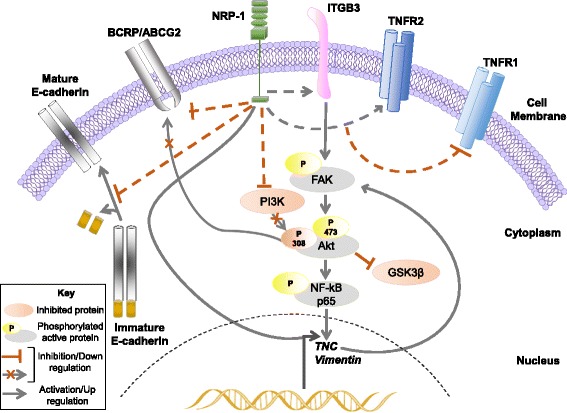
NRP-1/TNC/Integrin β3/FAK/Akt/NF-kB schematic signaling pathway. Schema to illustrate the identified mechanism by which NRP-1 activates TNC-dependent integrin β3 signaling via phosphorylation of FAK/Akt-473/NF-κB p65. In addition, NRP-1 upregulates TNFR2 expression and downregulates BCRP/ABCG2, TNFR1, and PI3K/Akt-308. Solid black lines indicate identified direct interactions in the pathway whereas dashed lines indicate indirect effects of NRP-1 overexpression via unidentified mechanisms. The key denotes the arrows and colors used in the illustration

A key downstream target of Akt signaling that is essential for Akt-induced oncogenic transformation is activation of NF-kB, which translocates to the nucleus and directly affects transcription of genes associated with tumorigenesis [34, 35]. We report that the increased tumorigenicity in vitro observed in NRP-1 overexpression is associated with increased activation of the p65 subunit of NF-κB. NF-κB is known to drive NRP-1-mediated EMT and migration in breast cancer [17]. Interestingly, NF-κB p65 was shown to promote *TNC* transcription in myeloid cells in an Akt-dependent pathway [36]. This evidence further strengthens the mechanistic pathway indicated in this report by which NRP-1 overexpression induces *TNC* transcription by activation of Akt/NF-κB signaling, and this may provide a positive feedback regulation via activation of ITGB3 (Fig. 8).

NRP-1 overexpression also triggered a downregulation in *APOD*, *ATF3* and *DDIT3* expression, all of which have been associated with an inverse regulation of tumourigenesis. ATF3 is known to function as a tumour suppressor in several cancer types [37–40]. Decreased ApoD glycoprotein expression has been associated with high grade progressive invasive breast cancer [41]. DDIT3 is a transcription factor that regulates stress response and has been shown to inhibit migration and induce genes involved in growth arrest and apoptosis [42, 43]. Interestingly, DDIT3 forms heterdimers with ATF3 to regulate stress-response related genes [44]. The role of two additional genes, *P2X6R* and *ACE* that were confirmed to be downregulated in BT-474 NRP-1 cells is controversial. P2X receptors are purinergic ion channel receptors whose role is contradictory, cell type-specific and dependent on ATP concentration but there is a general consensus of their function in cancer cell growth inhibition, especially P2X5R and P2X7R [45, 46]. The role of P2X6R in breast cancer has not been fully investigated, however it is likely that is has a common function as the other members of the P2X family in cancer growth inhibition. ACE, which was downregulated by NRP-1 in BT-474 cells has been reported to have pro-tumourigenic effects in various cancer types [47] although its homologue ACE2 has anti-tumour roles [48]. The role and interaction of these DEGs in association with NRP-1 need to be addressed in detail in future studies.

The role of NRP-1 in breast cancer-related chemoresistance has not been previously explored. A few reports indicated that NRP-1 downregulation or antagonism inhibited migration and enhanced chemosensitivity in non-small cell lung, kidney or prostate cancer [49] and osteosarcoma cells [50]. In pancreatic cancer cells, NRP-1 overexpression induced constitutive MAPK signaling and chemoresistance, and inhibited anoikis [51]. In this study, we report that NRP-1 inversely regulates ABCG2 expression and hence, plays a critical role in determining cellular response to chemotherapy. ABCG2 acts as an ATP-binding cassette efflux transporter for numerous anticancer agents including Adriamycin and has been implicated in eliciting multi-drug resistance [52]. High baseline expression of NRP-1 concomitant with low ABCG2 sensitizes chemo-naïve BT-474 NRP-1 cells to the effect of Adriamycin/Cyclophosphamide by decreasing NRP-1/ITGB3/FAK/Akt signaling concurrently with decreased tumorigenic behavior. In contrast, wild-type BT-474 cells have low baseline NRP-1/high ABCG2 where 4xAC treatment upregulates the NRP-1/ITGB3 pathway and thus promotes chemoresistance. Our proposition is strengthened by the observation of high baseline NRP-1 in untreated TNBC cells such as MDA-MB-231 as well as in TNBC tumors [4], which are known to be more sensitive to chemotherapy compared to other molecular subtypes [53]. Although increased NRP-1 expression in wild-type BT-474 4xAC cells is associated with lower expression of ABCG2, these cells display a more resistant phenotype, thus indicating that NRP-1 is a more predictive marker of chemoresistant breast cancer than ABCG2. The direct correlation of Phospho-Akt 308 with ABCG2 but inverse correlation with NRP-1 and ITGB3 provides a mechanistic explanation of ABCG2 regulation since Phospho-Akt 308 is known to regulate the cellular localization of ABCG2, whereby inhibition of the PI3K/Akt pathway is associated with ABCG2 degradation [54].

The cellular response on addition of Paclitaxel in 4xAC + 4xPAC resistant cells, however, showed variability. Treatment with Paclitaxel reversed the increased proliferation triggered by 4xAC in BT-474 wild-type cells but did not affect the proliferation of BT-474 NRP-1 resistant variants. Thus, in the context of proliferation it seems that BT-474 4xAC cells are more sensitive to Paclitaxel in comparison to NRP-1 overexpressing BT-474 NRP-1 4xAC cells. This observation is supported by reports stating that Paclitaxel triggers the most effective response in highly proliferating cells and anthracycline-resistant cancers [55, 56], which is characteristic of the BT-474 4xAC cells. On the other hand, the clonogenic potential of BT-474 4xAC + 4xPAC cells was similar to its 4xAC counterpart, whereas in the NRP-1 overexpressing cells 4xAC + 4xPAC treatment reversed the decreased clonogenic ability triggered by 4xAC but synergistically reduced migration compared to untreated and 4xAC cells. Thus, Paclitaxel seems to differentially affect various tumorigenic properties and hence, resistance to Paclitaxel, especially in combination with AC – a commonly used treatment regimen in breast cancer – needs to be further investigated.

## Conclusions

In summary, we indicate for the first time the global transcriptomic changes triggered by NRP-1 overexpression in breast cancer. We report the involvement of NRP-1 in activating the ITGB3 pathway via FAK/Akt-473/NF-κB p65 that is dependent on *TNC* expression, in addition to the upregulation of pro-survival TNFR2 and downregulation of TNFR1, PI3K/Akt-308, (Fig. 8). Moreover, we highlight the functional role NRP-1 plays in breast cancer resistance by attenuating ABCG2. These observations support the development of targets for the NRP-1/TNC/Integrin β3 axis and the validation of NRP-1 as a predictive biomarker for chemoresistance in breast cancer patients.

## Additional files

Additional file 1: **Table S1.** List of primary antibodies used. **Table S2.** List of RT-qPCR primer sequences. **Table S3.** List of significant upregulated DEGs. **Table S4.** List of significant downregulated DEGs. **Table S5.** List of shortlisted DEGs for confirmation by RT-qPCR. (DOCX 27 kb)

Additional file 2: **Figure S1.** Cellular response to NRP-1 overexpression. NRP-1 overexpression significantly increased **A**, clonogenicity, **B**, proliferation but **C**, did not significantly alter invasion through a basement membrane although there was a trend to increase. Images are representative of 3 independent experiments, all with comparable outcome. Graphs represent the mean ± SEM of 3 or more independent experiments. Statistical analysis using independent samples t-test, *p* value < 0.05 considered as statistically significant *** *p* < 0.001. (PPTX 35 kb)

## Abbreviations

AC: Adriamycin/Cyclophosphamide
BCRP: Breast Cancer resistant Protein
CPM: Counts Per Million
DEG: Differentially Expressed Gene
ECM: Extracellular Matrix
EGFR: Epidermal Growth Factor Receptor
EMT: Epithelial-to-Mesenchymal Transition
FAK: Focal Adhesion Kinase
FDR: False Detection rate
GO: Gene Ontology
GSK3β: Glycogen Synthase Kinase 3 beta
ITGB3: Integrin beta 3
MAPK: Mitogen-activated Protein Kinase
NF-kB: Nuclear Factor kappa B
NRP-1: Neuropilin-1
PAC: Paclitaxel
PI3K: Phosphatidyl Inositol-3 Kinase
TNC: Tenascin C
TNFR1/2: Tumor Necrosis Factor 1/2
