# Supplementary material for: Neuropilin-1 promotes the oncogenic Tenascin-C/integrin β3 pathway and modulates chemoresistance in breast cancer cells
Source: BMC Cancer. 2018 May 5;18:533. doi: 10.1186/s12885-018-4446-y (PMC5935908; doi:10.1186/s12885-018-4446-y)
Supplement: Supplementary file 1 — Table S1. List of primary antibodies used. Table S2. List of RT-qPCR primer sequences. Table S3. List of significant upregulated DEGs. Table S4. List of significant downregulated DEGs. Table S5. List of shortlisted DEGs for confirmation by RT-qPCR. (DOCX 27 kb) [file 12885_2018_4446_MOESM1_ESM.docx]

**Table S1. List of primary antibodies used**

| **Antibody** | **Company** |
| --- | --- |
| Anti-NRP-1 | Abcam |
| Anti-GAPDH | Cell Signalling Technology |
| Anti-E-cadherin | Cell Signalling Technology |
| Anti- β-catenin | Cell Signalling Technology |
| Anti-Integrin β3 | Cell Signalling Technology |
| Anti-Integrin β1 | Cell Signalling Technology |
| Anti-Integrin β4 | Cell Signalling Technology |
| Anti-Integrin β5 | Cell Signalling Technology |
| Anti-Phospho-FAK Tyr 397 | Abcam |
| Anti-FAK | Abcam |
| Anti-PI3K | Cell Signalling Technology |
| Anti-Phospho-Akt Ser473 | Cell Signalling Technology |
| Anti-Phospho Akt Ser308 | Cell Signalling Technology |
| Anti-Akt | Cell Signalling Technology |
| Anti-Phospho GSK3β | Cell Signalling Technology |
| Anti-GSK3β | Cell Signalling Technology |
| Anti-Phospho-NF-kB p65 | Cell Signalling Technology |
| Anti-NF-kB p65 | Cell Signalling Technology |
| Anti-Cyclin D1 | Abcam |
| Anti-p27 | Abcam |
| Anti-TNFR2 | Cell Signalling Technology |
| Anti-TNFR1 | Cell Signalling Technology |
| Anti-BCRP | Abcam |

**Table** **S2. List of RT-qPCR primer sequences**

| NRP-1 F | CCAGGTCGAATCCGATCCT | NM_003873.5 |
| --- | --- | --- |
| NRP-1 R | CGCTGTCGGTGTAAAAAACCA |  |
| Actin, beta F | CCAACCGCGAGAAGATGA | NM_001101.3 |
| Actin, beta R | CCAGAGGCGTACAGGGATAG |  |
| GUSB F | GAAAATACGTGGTTGGAGAGCTCA | NM_000181.3 |
| GUSB R | CCGAGTGAAGATCCCCTTTTTA |  |
| Vimentin F | AATGACCGCTTCGCCAACT | NM_003380.3 |
| Vimentin R | ATCTTATTCTGCTGCTCCAGGAA |  |
| CTNND2 F | TCACAGGTGCTGCGTAACG | NM_001332.3 |
| CTNND2 R | CTCTCCGGCCGAACTAACATT |  |
| CTTNBP2 F | TCTCCAGATGATGCAGAACTACCT | NM_033427.2 |
| CTTNBP2 R | TGGGCCGTGGAAAATGAC |  |
| GAS2L3 F | CCCCAAGGCCAAGGTTATTC | NM_174942.2 |
| GAS2L3 R | AGTGTGGACTCGGGCAGATC |  |
| GPC6 F | GGGCACAGCAAAGCCAGATA | NM_005708.3 |
| GPC6 R | TGGTTGGTGAGCCCATCAT |  |
| LRRK2 F | CGAGACCTGAAACCCCACAA | NM_198578.3 |
| LRRK2 R | CAATGCCGTAGTCAGCAATCTT |  |
| PLS3 F | TGGCATTGGAGGGCAAGA | NM_005032.6 |
| PLS3 R | GCTGCCAGACTAAAGCTAAAGTCA |  |
| SOCS2 F | CCTTTATCTGACCAAACCGCTCTA | NM_003877.4 |
| SOCS2 R | AATGGTGAGCCTACAGAGATGCT |  |
| TARP F | GGAACATACCTTTGTCTTCTTGAGAAAT | NM_001003799.1 |
| TARP R | GAATCGTGTTGCTCTTCTTTTCTTG |  |
| TGIF2-C20orf24 F | CCCAAGGAGTCGGTGAAGATC | NM_001199535.1 |
| TGIF2-C20orf24 R | GCGTTGTAGCGGTGCAAGT |  |
| TNC F | GGAACCAGGACAGGAGTACAATG | NM_002160.3 |
| TNC R | CACGTGCGGGCTTGCT |  |
| ACE F | GCACCCAGGCCAGGAAGT | NM_000789.3 |
| ACE R | CCTTCTTTATGATCCGCTTGATAGT |  |
| APOD F | CGGCAGAGGGACAAGCATT | NM_001647.3 |
| APOD R | TCCTGCACCGGAGGATTG |  |
| ATF3 F | GACGGAGTGCCTGCAGAAA | NM_001674.3 |
| ATF3 R | CCTTCAGTTCAGCATTCACACTTT |  |
| DDIT3 F | GGGAGCTGGAAGCCTGGTAT | NM_001195053.1 |
| DDIT3 R | CCCCCATTTTCATCTGAAGACA |  |
| HSPA6 F | AGGGTGAGAGGGCCATGAC | NM_002155.4 |
| HSPA6 R | CAGGAGGGATGCCACTGAGT |  |
| MOV10L1 F | CTGCAGGTTCGAGGAGATAGTTATT | NM_018995.2 |
| MOV10L1 R | GATGTCTTCTCCGTCTCTGCAAT |  |
| P2RX6 F | AGGCCAGTGTGTGGTGTTCA | NM_005446.4 |
| P2RX6 R | GGGCACCAACTCCAGATCTC |  |
| RIMBP3 F | GGGAGCACCCTATTGGAATTC | NM_005446.4 |
| RIMBP3 R | GACTCACCACAGAGTGACATGGTT |  |
| TFCP2L1 F | AGTGGCTTCACCGCAACAG | NM_014553.2 |
| TFCP2L1 R | CACCTGAGAAGCTGGCAAAGA |  |
| TNF F | TGCTCCTCACCCACACCAT | NM_000594.3 |
| TNF R | GGAGGTTGACCTTGGTCTGGTA |  |
| SOX2 F | TGCGAGCGCTGCACAT | NM_003106.3 |
| SOX2 R | TCATGAGCGTCTTGGTTTTCC |  |
| SOX6 F | CCAGAGCGCCGCAAAG | NM_017508.2 |
| SOX6 R | TCAAGCTTCTTCTGTTTCAGTGTGT |  |

**Table S3. List of significant upregulated DEGs.**

| **DEG- Upregulated** | **Replicate 1 fold-change** | **Replicate 2 fold-change** |
| --- | --- | --- |
| TGIF2-C20orf24 | 8.791162889 | 7.906890596 |
| ANXA2P2 | 7.357552005 | 2.5360529 |
| TARP | 6.389452089 | 5.554588852 |
| PCSK9 | 5.489384841 | 3.831952286 |
| CA8 | 5.426264755 | 2.684498174 |
| CTTNBP2 | 4.169925001 | 3.614709844 |
| NRP1 | 4.03562391 | 2.934904972 |
| BPIFA4P | 3.958693026 | 2.088853391 |
| SCGN | 3.66801824 | 2.11321061 |
| TNC | 3.284814922 | 3.723616041 |
| NRK | 3.133855747 | 4.169925001 |
| GPC6 | 2.974004791 | 3.608809243 |
| CLCA2 | 2.964771512 | 3.256040159 |
| LRRK2 | 2.887525271 | 5.247927513 |
| GAS2L3 | 2.795415309 | 2.039819318 |
| SOX6 | 2.754887502 | 2 |
| BASP1 | 2.679200922 | 2.831684251 |
| TUBA1A | 2.513339573 | 2.009114987 |
| CPVL | 2.407503827 | 2.181688451 |
| CTNND2 | 2.085487899 | 2.153156788 |
| SOCS2 | 2.042041771 | 2.037868186 |
| PLS3 | 2.010014892 | 2.248147587 |
| COL12A1 | 2 | 2.166318262 |

**Table S4. List of significant downregulated DEGs.**

| DEG- Downregulated | Replicate 1 fold-change | Replicate 2 fold-change |
| --- | --- | --- |
| CTSF | -2.024045474 | -2.192782944 |
| KCNF1 | -2.02666795 | -2.284208429 |
| ATF3 | -2.029630964 | -3.457704874 |
| ODF3B | -2.044007182 | -2.921191159 |
| CSTA | -2.06161319 | -5.192516992 |
| WNT5B | -2.091147888 | -3.816692787 |
| DDIT3 | -2.112891013 | -2.287016671 |
| MCHR1 | -2.133483005 | -2.639410285 |
| ACE | -2.161463423 | -5.357552005 |
| C2CD4B | -2.246311048 | -2.992466327 |
| SAMD14 | -2.247927513 | -3.191141487 |
| TMEM61 | -2.251918374 | -2.337869639 |
| SLC13A3 | -2.253756592 | -2.93657462 |
| TTYH1 | -2.267772325 | -6.357552005 |
| IFITM10 | -2.281319759 | -2.362311509 |
| DQX1 | -2.284208429 | -3.067744607 |
| CYP2B7P | -2.309059293 | -2.162109181 |
| PANX2 | -2.312882955 | -2.379538818 |
| STRC | -2.331843564 | -3.517275693 |
| GPR124 | -2.342392197 | -2.142957954 |
| PATL2 | -2.343301746 | -2.841302254 |
| HSPA6 | -2.415037499 | -6.326838009 |
| PLB1 | -2.563900885 | -2.531720479 |
| IL20RA | -2.574470127 | -2.033947332 |
| MYO15A | -2.632268215 | -2.925999419 |
| SLC43A1 | -2.689713312 | -2.116305163 |
| WBSCR27 | -2.692630795 | -3.469681715 |
| LCN12 | -2.748268243 | -3.142444265 |
| KLHDC7B | -2.769771739 | -5.373300197 |
| KCNU1 | -2.892535303 | -2.748815312 |
| ANK1 | -2.901819606 | -2.139930261 |
| DCST2 | -2.906890596 | -2.358686645 |
| TFCP2L1 | -2.935459748 | -2.061400545 |
| TMEM92 | -2.978766166 | -2.72022233 |
| SCNN1B | -3.072900547 | -3.515357033 |
| ARHGEF25 | -3.098032083 | -2.146841388 |
| TEX19 | -3.143835773 | -2.158697746 |
| TNXB | -3.195550809 | -3.599317794 |
| UNC5B-AS1 | -3.197087332 | -5.40849735 |
| GUCY2EP | -3.203283598 | -3.200139614 |
| P2RX6 | -3.28757659 | -3.152003093 |
| MYH15 | -3.292781749 | -2.292781749 |
| MOV10L1 | -3.337869639 | -6.62935662 |
| LCN2 | -3.43898596 | -4.262475596 |
| SERPINA3 | -4.0191151 | -4.472584763 |
| APOD | -4.200139614 | -7.584962501 |
| RIMBP3 | -4.906890596 | -5 |
| ASIC4 | -4.942514505 | -5.64385619 |
| B3GAT1 | -5.044394119 | -4.874469118 |
| SERPINA6 | -5.187352073 | -3.284614936 |
| KRT6B | -5.459431619 | -3.325442579 |
| ABCC6 | -5.510961919 | -2.347923303 |
| STON1-GTF2A1L | -5.523561956 | -5.781359714 |
| FIBIN | -6.087462841 | -5.700439718 |
| LRRC32 | -6.341630009 | -3.736450254 |
| SRRM3 | -7.118941073 | -3.772589504 |
| PDZK1IP1 | -7.607330314 | -7.483815777 |
| KLK8 | -7.74819285 | -3.596367264 |
| ANKRD29 | -8.129283017 | -2.38932738 |
| TNF | -8.581200582 | -3.517575661 |
| TMX2-CTNND1 | -9.221587121 | -2.176426567 |

**Table S5. List of shortlisted DEGs for confirmation by RT-qPCR**

| **Selected Upregulated Genes** | **Selected Downregulated Genes** |
| --- | --- |
| LRRK2 | ACE |
| PLS3 | APOD |
| TNC | ATF3 |
| GAS2L3 | DDIT3 |
| CTNND2 | HSP70 |
| TGIF2-C20orf24 | MOV10L1 |
| SOCS2 | P2RX6 |
| GPC6 | RIMBP3 |
| SOX6 | TFCP2L1 |
| TARP | TNF |
| CTTNBP2 | SOX2 |
