# Supplementary material for: Neuropilin-1 promotes the oncogenic Tenascin-C/integrin β3 pathway and modulates chemoresistance in breast cancer cells
Source: BMC Cancer. 2018 May 5;18:533. doi: 10.1186/s12885-018-4446-y (PMC5935908; doi:10.1186/s12885-018-4446-y)
Supplement: Supplementary file 2 — Figure S1. Cellular response to NRP-1 overexpression. NRP-1 overexpression significantly increased A, clonogenicity, B, proliferation but C, did not significantly alter invasion through a basement membrane although there was a trend to increase. Images are representative of 3 independent experiments, all with comparable outcome. Graphs represent the mean ± SEM of 3 or more independent experiments. Statistical analysis using independent samples t-test, p value < 0.05 considered as statistically significant *** p < 0.001. (PPTX 35 kb) [file 12885_2018_4446_MOESM2_ESM.pptx]

## Slide 1
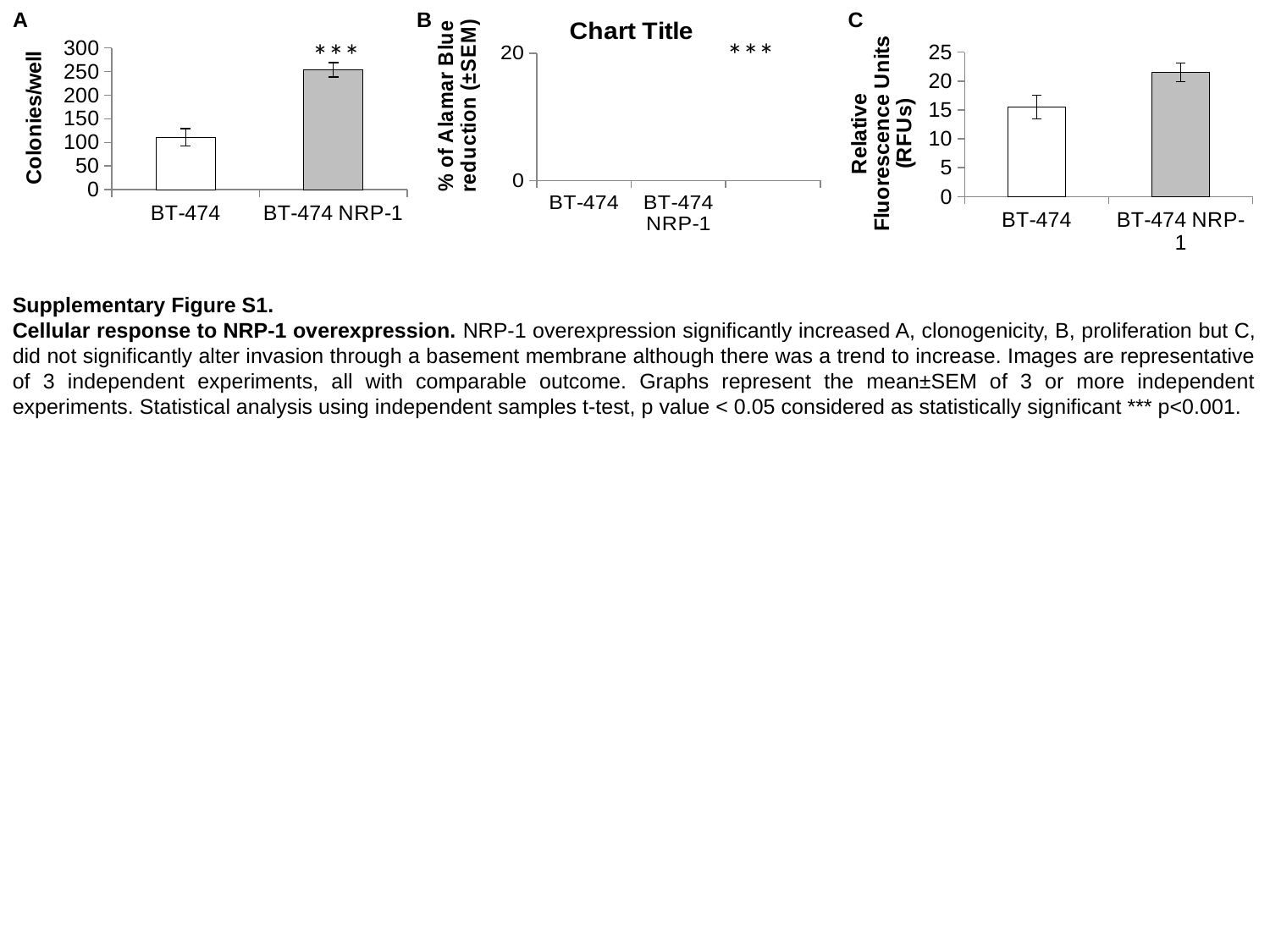

A
B
### Chart:
| Category | |
|---|---|
| BT-474 | 62.08375971416956 |
| BT-474 NRP-1 | 90.1484194232929 |***
C
### Chart
| Category | Average |
|---|---|
| BT-474 | 15.5 |
| BT-474 NRP-1 | 21.5 |
### Chart
| Category | Clonogenic assay |
|---|---|
| BT-474 | 110.5 |
| BT-474 NRP-1 | 253.6666666666667 |***
Supplementary Figure S1.
Cellular response to NRP-1 overexpression. NRP-1 overexpression significantly increased A, clonogenicity, B, proliferation but C, did not significantly alter invasion through a basement membrane although there was a trend to increase. Images are representative of 3 independent experiments, all with comparable outcome. Graphs represent the mean±SEM of 3 or more independent experiments. Statistical analysis using independent samples t-test, p value < 0.05 considered as statistically significant *** p<0.001.
